# Quitting smoking after a cancer diagnosis is associated with high-risk neutrophil-to-lymphocyte ratio among tobacco use-related cancer survivors

**DOI:** 10.1038/s41598-023-27624-1

**Published:** 2023-02-16

**Authors:** You Lu, Katherine Kwong, James Wells, Andrea Edwards, Zhong Chen, Tung-Sung Tseng, Kun Zhang

**Affiliations:** 1 Department of Physics and Computer Science, Xavier University of Louisiana, New Orleans, LA USA; 2 Department of Human Development, Connecticut College, New London, CT USA; 3 Department of Physiology, Tulane University, New Orleans, LA USA; 4 Behavioral and Community Health Science, School of Public Health, Louisiana State University Health Science Center, 2020 Gravier Street, Room 213, New Orleans, LA 70112 USA; 5 Bioniformatics Core of Xavier NIH RCMI Center of Cancer Research, Xavier University of Louisiana, 1 Drexel Drive, 540 NCF Annex, New Orleans, LA 70125 USA

**Keywords:** Disease-free survival, Prognostic markers, Epidemiology

## Abstract

Quitting smoking could potentially minimize the risk of a high neutrophil-to-lymphocyte ratio (NLR) among tobacco use-related (TUR) cancer survivors. A total of 1263 TUR cancer survivors aged 20 to 85 years old were investigated using data from the National Health and Nutritional Examination Survey 1999–2018. The primary outcome was the NLR, which was defined as having two levels: high-risk (≥ 3) and low-risk (< 3). The association between smoking cessation time and a high-risk NLR level was analyzed using weighted logistic regression models. Overall, the current smoking rate of TUR cancer survivors was found to be 21.7%. Older age (75 years above), gender and respiratory-related cancers are covariables associated with high risk of NLR levels for individual who identified as Non-Hispanic White (NHW). Non-Hispanic Black (NHB) (n = 27) who quit smoking after a cancer diagnosis were associated with the highest risk of a high NLR (OR 4.83, 95% CI 1.40–16.61, *p* = 0.01) compared to NHB nonsmokers (n = 139). These findings suggest that the risk of a high NLR level is strongly associated with the time of smoking cessation in NHB TUR cancer survivors. As a result, NHB TUR cancer survivors should quit smoking as soon as possible because the benefits of quitting smoking were observed over the 5 year period following smoking cessation.

## Introduction

Smoking cessation is an effective way to increase the overall survival (OS) rate and reduce disease-specific mortality in cancer survivors. It is an interventional approach that has been extensively advocated for in previous studies^1,2^. For individuals who were diagnosed with tobacco use-related (TUR) cancer, such as lung, bladder, kidney, colon, etc.^3,4^, the disease-free period and cancer recurrence rate were sensitive to their smoking cessation time. A previous study examined the association between smoking status after diagnosis and survival of 206 non-small cell lung cancer patients and found that individuals who quit smoking after their cancer diagnosis maintained a better performance status at six and twelve months compared to individuals who continued to smoke^5^. The association between continuing to smoke after a cancer diagnosis and both an increased cancer recurrence rate and potential cancer progression has been consistently reported from other TUR cancer studies^6^. Shiels, MS, et al., compared the association between the risk of developing a second TUR cancer and cigarette smoking before first cancer diagnosis by pooling data from five cohort studies (conducted between 1994 and 2001), which included 2552 stage I lung cancer patients, 6386 bladder cancer patients, 3179 kidney cancer patients, and 2967 head/neck cancer patients. They found that, for the survivors of these selected cancers that smoked, currently smoking more than 20 cigarettes per day was associated with an increased risk of a second TUR cancer for stage I lung (hazard ratio [HR] = 3.26), bladder (HR = 3.67), kidney (HR = 5.33), and head/neck (HR = 4.45) cancer survivors compared with non-smokers^7^. In addition, one study focused on 2933 colon cancer survivors and found that the disease-specific (HR = 1.30) and the all-cause mortality (HR = 1.51) rates were significantly higher if the colon cancer survivors that continued smoking compared with never-smokers^8^.

Another study found continued tobacco smoking after a cancer diagnosis was associated with adverse outcomes among 868 cancer survivors from the Measuring Your Health study, a community-based survey cohort of cancer patients who registered across four Surveillance, Epidemiology, and End Results cancer registries^9^. This study observed that, within a year of diagnosis, individuals who were diagnosed with a TUR cancer were three times more likely to quit smoking compared to patients with non-TUR cancers. Additionally, a recently published cohort study investigated over 1124 patients with newly diagnosed non-small cell lung cancer (NSCLC) between 2010 and 2016. For these cancer survivors who were defined as current smoker, 23% of them quit smoking within three months of diagnosis. In this study, however, the observed association between a lower risk of mortality and smoking cessation after NSCLC diagnosis was not statistically significant^10^.

As an independent inflammation biomarker, the neutrophil-to-lymphocyte ratio (NLR) level is considered a prognostic factor that is influenced by smoking cessation in various cancer prevention and cancer management programs^11,12^. Typically, individuals who continued smoking after a diagnosis of TUR cancer would have high-risk NLR levels, further indicating a worse OS rate. For instance, bladder cancer survivors with NLR levels over 2.5 who continued smoking after a cancer diagnosis were associated with a cancer recurrence rate 2.2 times higher than former smokers who ceased smoking before a cancer diagnosis^13^. However, other risk factors, such as race, chronic disease status, etc., should also be considered when comparing different smoking cessation times with the high-risk NLR levels in TUR cancer survivors. Non-Hispanic Black (NHB) patients usually have lower mean neutrophil counts, but similar lymphocyte numbers compared to Non-Hispanic White (NHW) patients^14,15^. This racial difference in immune cell composition indicates that using multiple cut-off values for NLR is recommended when estimating health-related outcomes from a cohort consisting of multiple racial groups^16^. Additionally, individuals with multiple chronic diseases, e.g. diabetes and arthritis, have higher NLR levels than disease-free individuals^17^.

Cancer diagnosis offer a flexible time for implementation tobacco-related smoking cessation intervention, however patients with cancer diagnosis are less motivated to quit cigarette smoking or engage with treatments^18^. Barriers for quit smoking after cancer diagnosis including nicotine addition, lacking self-efficacy, social supports, and etc. have been widely explored^19–21^. Still how high-risk of NLR levels associated with quit smoking at the certain time point after cancer diagnosed remain unclear.

The purpose of this study is to first, the association between different smoking quitting times before and after a cancer diagnosis and the risk of high NLR levels among tobacco use-related cancer survivors will be explored. Secondary, a logistic regression model to examine the effects of selected covariables in terms of high-risk of NLR levels will be developed. In addition, how racial disparity affects the level of NLR will be examined.

## Methods

### Study design

We combined and extracted ten consecutive 2-year survey cycle datasets (1999–2018) from the US Centers for Disease Control NHANES dataset for this study. Based on the determination of tobacco use-related cancers from the Centers for Disease Control and Prevention (CDC)^3^ and findings from a study that was conducted by the US National Cancer Institute^4^, we conducted a secondary data analysis on fourteen tobacco use-related cancer types, including: bladder, kidney, colon, esophagus, mouth, larynx, stomach, liver, lung, pancreatic, rectal, prostate, and cervical cancer, and lymphoma/Hodgkin's disease. Adult cancer survivors between the ages of 20 and 85 years old with a completed response to the questions on smoking patterns and a recorded NLR level were included in this analysis. Tobacco use data was collected from all survey participants at 20 years of age and older, with a subset of questions during the Mobile Examination Center (MEC) interview. A multistage, stratified, probability-cluster sampling method was used during the NHANES data collection, under the supervision of the National Center for Health Statistics of the CDC^22^. An in-person, face-to-face interview was conducted for qualified participants at their homes by trained staff. Individual demographic and health-related information was collected via examination. The collected blood and urine samples from the participants were sent to laboratories for further analysis^22^. The survey protocol was revised and approved by the National Center for Health Statistics Research Ethics Review Board^23^. A paper-based informed consent form was signed by every participant before the data collection process was initiated.

### Measurements

We used two NHANES questions to extract data on tobacco use-related cancers: “Have you ever been told by a doctor or other health professional that you had cancer or a malignancy of any kind?” and “What kind of cancer was it?” Participants with TUR cancer types were identified based on their primary cancer diagnosis. Participants with more than one cancer type were excluded from this analysis. For all individuals diagnosed with TUR cancer, their smoking status was divided into nonsmoker (NS), former smoker one (FS1), former smoker two (FS2), former smoker three (FS3), and current smoker (CS) based on the following questions: “Have you smoked at least 100 cigarettes in your entire life?” and “Do you now smoke cigarettes?” A NS was defined as a respondent who reported that they smoked < 100 cigarettes in their lifetime; CS was defined as respondents who reported smoking ≥ 100 cigarettes in their entire lifetime and were currently smoking every day or some days^24^. We defined FS1 as participants who quit smoking 5 or more years before a cancer diagnosis; FS2 was defined as participants who quit smoking less than 5 years before a cancer diagnosis^25^; and FS3 was defined as participants who quit smoking after a cancer diagnosis. In addition, the question regarding “age when *X* cancer was first diagnosed,” where “X” represents any of the tobacco use-related cancers mentioned previously, was used in analyzing when individuals had quit smoking. The demographic information of included participants included age (< 45, 45–54, 55–64, 65–74, 75–85), race/ethnicity (NHW, NHB), gender, and education (less than high school [< High School], and high school and above [≥ High School]). Individuals without chronic bronchitis, coronary heart disease, or diabetes were considered chronic disease-free (CD free). In addition, Body-Mass-Index (BMI) was classified as underweight/normal for those with a BMI < 25, and overweight/obese for those with a BMI ≥ 25^26^. Fourteen cancer types were stratified as either respiratory cancers (RCs) (lung and larynx) or non-respiratory cancers (NRCs) (bladder, kidney, colon, esophagus, mouth, stomach, liver, pancreatic, rectal, prostate, and cervical cancer, and lymphoma/Hodgkin's disease). According to the cancer diagnosis timeline, six levels of Year After a Cancer Diagnosis (YACD) were defined as 0, <1, 1.00–1.99, 2.00–4.99, 5.00–9.99, ≥ 10.00.

### Ethics

The procedures to obtain human data have been performed in accordance with the Declaration of Helsinki. All methods were carried out in this study was reviewed and approved by the Institutional Review Board of Xavier University of Louisiana. The study used publicly available datasets that meet the federal regulation at 46 CFR 46.102.

### Outcomes

To calculate the NLR level, the neutrophil number (1000 cell/µL) and the lymphocyte number (1000 cell/µL) were used from complete blood count measurements, which were obtained from the NHANES laboratory datasets^27^. NLR is defined as the total number of neutrophils divided by the total lymphocyte number. For cancer survivors, a high NLR level is associated with a poor OS rate. In this study, the mean NLR was 2.62, and, for our analysis, we defined a high-risk NLR level as NLR ≥ 3, and a low-risk NLR level was defined as NLR < 3^12,28^.

### Statistical analysis

Participants’ demographic, smoking behavior, and clinical features were summarized using descriptive statistics based on the different NLR levels (low and high). The association of these factors with the NLR level was tested using the Rao-Scott Chi-square test for categorical variables and Fisher’s exact test for small samples. A one-way ANOVA test was applied to examine differences for continuous variables. All analyses were performed in R (svydesign) using the “survey” package^29^ to create the weighted analysis groups for displaying percentages (%) (svyciprop) and means with standard error of the mean (svymean). Survey-weighted univariable logistic regression models (svyglm) were applied to examine the association between smoking status and risk of high NLR level in cancer survivors. We included the suggested covariables: age, gender, race, BMI, CD free status, cancer type, education level, and YACD to construct the multivariable logistic regression model^30–38^. All variables had no multicollinearity in the model. We followed the weighting instructions provided on the CDC website^39^. All analyses were conducted using R version 3.6.3 with the “survey” and “dplyr” packages. All tests were 2-sided, and a *p* value < 0.05 was considered statistically significant for all tests.

## Results

A total of 1263 cancer survivors between the ages of 20 and 85 years old were included from NHANES 1999–2018. Overall, 35.5% of participants were nonsmoker (smoking < 100 cigarette in their lifetime); 27.5% of participants quit smoking 5 or more years before a cancer diagnosis; 6.1% of participants quit smoking less than 5 years before TUR cancer diagnosis; 9.2% of participants quit smoking after a cancer diagnosis, and the current smoking rate among cancer survivors was 21.7%. Our study found 28.2% of cancer survivors (n = 382) had high-risk NLR levels. Table 1 provides weighted percentages and raw sample sizes for demographics and smoking status by high-risk (≥ 3) and low-risk (< 3) NLR level. Cancer survivors in the low-risk NLR group had a longer mean years of survival after a cancer diagnosis compared to individuals in the high-risk group (11.2 ± 0.4, 9.1 ± 0.5, *p* < 0.01); additionally, the low-risk NLR group had a younger mean age compared to the high-risk NLR group (67.7 ± 0.8, 60.2 ± 0.7, *p* < 0.01). NLR across the high-risk and low-risk groups was distributed differently in terms of smoking status. Race, gender, age, CD free status, and cancer type were statistically significant factors and associated with the varied distribution of NLR level in the studied population. Cancer survivors with a lower risk of high NLR levels were found in the NHW, female, age ≥ 45, CD free, and NRC groups compared to their counterparts.

**Table 1:** Characteristics of 1263 cancer survivors with different NLR risks using NHANES 1999–2018 Data.

Characteristics	Overall	NLR < 3	NLR ≥ 3	*p* value
Total, n (%)	1263 (100)	881 (71.8)	382 (28.2)	
Mean NLR (± SEM)	2.62 (± 0.04)	1.93 (± 0.02)	4.39 (± 0.09)	
Mean Age (± SEM), year	62.3 (± 0.6)	60.2 (± 0.7)	67.7 (± 0.8)	< 0.01^†^
Mean YACD (± SEM), year	10.6 (± 0.3)	11.2 (± 0.4)	9.1 (± 0.5)	< 0.01^†^
Smoking Status, n (%)				< 0.01^a^
NS	457 (35.5)	337 (74.3)	120 (25.7)	
FS1	372 (27.5)	243 (64.9)	129 (35.1)	
FS2	83 (6.1)	47 (59.0)	36 (41.0)	
FS3	115 (9.2)	75 (71.3)	40 (28.7)	
CS	236 (21.7)	179 (80.5)	57 (19.5)	
Race, n (%)				< 0.01^a^
NHW	915 (88.7)	601 (70.7)	314 (29.3)	
NHB	348 (11.3)	280 (80.9)	68 (19.1)	
Gender, n (%)				< 0.01^a^
Male	828 (54.8)	541 (65.2)	287 (34.8)	
Female	435 (45.2)	340 (79.9)	95 (20.1)	
Age, n (%)				< 0.01^a^
< 45	144 (17.1)	122 (85.5)	22 (14.5)	
45–54	99 (12.4)	78 (78.1)	21 (21.9)	
55–64	200 (17.5)	161 (82.4)	39 (17.6)	
65–74	318 (24.0)	218 (66.7)	100 (33.3)	
75–85	502 (28.9)	302 (58.9)	200 (41.1)	
CD Free, n (%)				0.01^a^
Yes	800 (65.4)	574 (74.6)	226 (25.4)	
No	463 (34.6)	307 (66.6)	156 (33.4)	
BMI, n (%)				0.07^a^
Under/Normal	398 (30.6)	260 (67.4)	138 (32.6)	
Overweigh/Obese	865 (69.4)	621(73.8)	244 (26.2)	
Education Level, n (%)				0.89^a^
< High School	318 (18.0)	212 (28.5)	106 (71.5)	
≥ High School	945 (82.0)	669 (28.1)	276 (71.9)	
Cancer Types, n (%)				< 0.01^a^
RCs	78 (6.7)	40 (53.7)	38 (46.3)	
NRCs	1185 (93.3)	841 (73.1)	344 (26.9)	
YACD, n (%)				0.12^a^
0.00–1.99	195 (14.9)	125 (63.4)	70 (36.6)	
2.00–4.99	337 (24.6)	239 (72.6)	98 (27.4)	
5.00–9.99	281 (21.1)	203 (70.9)	78 (29.1)	
≥ 10.00	450 (39.4)	314 (75.0)	136 (25.0)	

^†^One-way ANOVA test found mean age is distributed differently between low-risk and high-risk NLR groups. ^a^Rao-Scott Chi-square test. *SEM* standard error of the mean. *NLR* Neutrophil-to-lymphocyte ratio. *CD* Chronic diseases. *BMI* Body mass index. *NHANES* National Health and Nutrition Examination Survey. *NHW* Non-Hispanic White. *NHB* Non-Hispanic Black. *NS* Nonsmoker. *FS1* Quit smoking 5 or more years before a cancer diagnosis. *FS2* Quit smoking less than 5 years before a cancer diagnosis. *FS3* quit smoking after a cancer diagnosis. *CS* continued smoking after a cancer diagnosis. *RCs* Respiratory cancers. *NRCs* Non-respiratory cancers. *YACD* Years after a cancer diagnosis.

No statistical difference in mean NLR levels was observed across the five smoking statuses (*p* = 0.29, Table 2). The average age of current smoker (49.6 ± 1.2) was younger than nonsmoker (65.6 ± 0.9), former smoker who quit smoking 5 or more years before a cancer diagnosis (70.0 ± 0.8), former smoker who quit smoking less than 5 years before a cancer diagnosis (63.1 ± 2.2), and former smoker who quit smoking after a cancer diagnosis (63.1 ± 2.0) (*p* < 0.04). Of note, the elderly between the ages of 75 and 85 years old were the least likely to be current smoker (4.9%) when compared to other age groups. For individuals who continued smoking, most of them were female (66.4%). Individuals who continued smoking had the lowest overweight or obese rate compared with nonsmoker, those that quit smoking 5 or more years before a cancer diagnosis, those that quit smoking less than 5 years before a cancer diagnosis, and those that quit smoking after a cancer diagnosis (55.0% vs.72.2%, 78.1%, 65.4%, and 69.1%). In addition, the current smoker group contained the highest ratio of individuals with less than a high school level education (24.2%). For individuals who quit smoking 5 or more years before a cancer diagnosis, most were identified as non-respiratory cancer patients (93.8%). For individuals who quit smoking less than 5 years of before a cancer diagnosis, over one-third of them were respiratory cancer survivors (31.7%). This percent of respiratory cancer survivors reduced to 11.9% for individuals who quit smoking after a cancer diagnosis and reduced even further to 5.6% for individuals that were current smoker. Interestingly, individuals who quit smoking after a cancer diagnosis had the largest proportion of survivors who had a longer cancer diagnosis time (≥ 10 years) compared to nonsmoker, those that quit smoking 5 or more years before a cancer diagnosis, those that quit smoking less than 5 years before a cancer diagnosis, and current smoker (65.3%, 39.4%, 26.0%, 29.4%, and 48.4%).

**Table 2:** Factors associated with smoking status in cancer survivors.

Characteristic	NS	FS1	FS2	FS3	CS	*p* value
Mean NLR (± SEM)	2.55 (± 0.07)	2.83 (± 0.11)	3.18 (± 0.24)	2.69 (± 0.14)	2.34 (± 0.10)	0.29^†^
Mean Age (± SEM), year	65.6 (± 0.9)	70.0 (± 0.8)	63.1 (± 2.2)	63.1 (± 2.0)	49.6 (± 1.2)	< 0.04^†^
Mean YACD (± SEM), year	10.3 (± 0.6)	7.5 (± 0.4)	9.6 (± 1.1)	17.7 (± 1.3)	11.5 (± 0.7)	0.85^†^
Race, n(%)						0.12
NHW	318 (87.1)	287 (91.7)	59 (86.6)	88 (88.8)	163 (88.0)	
NHB	139 (12.9)	85 (8.3)	24 (13.4)	27 (11.2)	73 (12.0)	
Gender, n(%)						< 0.01^a^
Male	286 (51.9)	311 (78.1)	58 (61.3)	71 (41.8)	102 (33.6)	
Female	171 (48.1)	61 (21.9)	25 (38.7)	44 (58.2)	134 (66.4)	
Age, n(%)						< 0.01^b^
< 45	45 (16.1)	8 (4.6)	6 (13.0)	8 (9.7)	77 (39.0)	
45–54	29 (10.4)	8 (5.1)	10 (14.6)	13 (20.0)	39 (21.1)	
55–64	74 (19.1)	40 (13.9)	12 (13.9)	19 (22.8)	55 (18.3)	
65–74	101 (19.3)	120 (34.8)	28 (33.6)	23 (20.6)	46 (16.8)	
75–85	208 (35.1)	196 (41.6)	27 (24.9)	52 (27.0)	19 ( 4.9)	
CD Free, n(%)						0.08^a^
Yes	314 (72.2)	219 (61.0)	45 (59.9)	70 (58.8)	152 (63.9)	
No	143 (27.8)	153 (39.0)	38 (40.1)	45 (41.2)	84 (36.1)	
BMI, n(%)						< 0.01^b^
Under/Normal	134 (27.8)	85 (21.9)	34 (34.6)	40 (30.9)	105 (45.0)	
Overweigh/Obese	323 (72.2)	287 (78.1)	49 (65.4)	75 (69.1)	131 (55.0)	
Education Level, n(%)						< 0.01^b^
< High School	97 (13.2)	99 (19.5)	16 (11.2)	31 (22.0)	75 (24.2)	
≥ High School	360 (86.8)	273 (80.5)	67 (88.8)	84 (78.0)	161 (75.8)	
Cancer Types, n(%)						< 0.01^b^
RCs	7 (2.1)	26 (6.2)	22 (31.7)	10 (11.9)	13 (5.6)	
NRCs	450 (97.9)	346 (93.8)	61 (68.3)	105 (88.1)	223 (94.4)	
YACD, n(%)						< 0.01^b^
0.00 ~ 1.99	72 (15.8)	72 (19.3)	17 (14.6)	6 ( 6.5)	28 (11.4)	
2.00 ~ 4.99	122 (24.6)	116 (30.3)	14 (21.5)	23 (13.0)	62 (23.1)	
5.00 ~ 9.99	105 (20.2)	92 (24.4)	30 (34.6)	15 (15.2)	39 (17.1)	
≥ 10.00	158 (39.4)	92 (26.0)	22 (29.4)	71 (65.3)	107 (48.4)	

^†^One-way ANOVA test found mean age is distributed differently between low-risk and high-risk NLR groups. ^a^Rao-Scott Chi-square test. ^b^Fisher’s exact test. *SEM* Standard error of the mean. *NLR* Neutrophil-to-lymphocyte ratio. *CD* Chronic diseases. *BMI* Body mass index. *NHW* Non-Hispanic White. *NHB* Non-Hispanic Black. *NS* Nonsmoker. *FS1* Quit smoking 5 or more years before a cancer diagnosis. *FS2* Quit smoking less than 5 years before a cancer diagnosis. *FS3* quit smoking after a cancer diagnosis. *CS* Continued smoking after a cancer diagnosis. *RCs* Respiratory cancers. *NRCs* Non-respiratory cancers. *YACD* Years after a cancer diagnosis.

The weighted logistic regression model for cancer survivors with a high-risk NLR level was computed and the results can be found in Table 3. In the unadjusted analysis, smoking status, race, gender, age, CD free status, cancer type, and YACD were independent variables that contributed to NLR level. After adjusting for covariate variables, we found female cancer survivors had lower odds of high-risk NLR compared to males (OR 0.63, CI 0.42–0.94, *p* = 0.02). Individuals who were diagnosed with non-respiratory cancers had lower odds of high-risk NLR compared to respiratory cancer survivors (OR 0.49, CI 0.27–0.88. *p* = 0.01). Individuals 75 years of age or older had higher odds of high-risk NLR compared to cancer survivors who are younger than 45 years of age (OR 2.81, CI 1.36–5.79, *p* < 0.01). No significant association between smoking status and the risk of high NLR levels in TUR cancer survivors was observed. NHB survivors had lower odds of high-risk NLR compared to NHW survivors (OR 0.56, CI 0.38–0.82, *p* < 0.01). We observed race interacts with smoking status and affects the risk of NLR level only in individuals who quit smoking after a cancer diagnosis (*p* = 0.03) and current smoker (*p* = 0.01). Further stratified analysis of the smoking statuses associated with high-risk NLR levels regarding NWB and NHW cancer survivors is needed.

**Table 3:** Factors associated with a high-risk NLR level (NLR ≥ 3).

Characteristic	Unadjusted OR	95% CI	*p* value	Adjusted OR	95% CI	*p *value
**Smoking status**						
NS (ref)	1			1		
FS1	1.56	1.02–2.37	0.03	1.16	0.74–1.81	0.51
FS2	2.00	1.13–3.55	0.01	1.53	0.82–2.89	0.18
FS3	1.16	0.66–2.04	0.59	1.16	0.66–2.04	0.58
CS	0.69	0.42–1.15	0.16	0.95	0.51–1.77	0.86
**Race**						
NHW (ref)	1			1		
NHB	0.56	0.41–0.79	< 0.01	0.56	0.38–0.82	< 0.01
**Gender**						
Male (ref)	1			1		
Female	0.47	0.32–0.67	< 0.01	0.63	0.42–0.94	0.02
**Age**						
< 45 (ref)	1			1		
45–54	1.65	0.66–4.12	0.27	1.48	0.57–3.84	0.41
55–64	1.25	0.57–2.72	0.55	1.04	0.46–2.36	0.92
65–74	2.94	1.48–5.83	< 0.01	1.92	0.91–4.05	0.08
75–85	4.11	2.21–7.65	< 0.01	2.81	1.36–5.79	< 0.01
**CD free**						
Yes (ref)	1			1		
No	1.47	1.06–2.03	0.01	1.34	0.95–1.89	0.08
**BMI**						
Under/normal (ref)	1			1		
Overweight/obese	0.73	0.52–1.03	0.07	0.74	0.50–1.08	0.11
**Education level**						
< High school (ref)	1			1		
≥ High school	0.97	0.70–1.36	0.89	1.05	0.74–1.50	0.77
Cancer types						
RCs (ref)	1			1		
NRCs	0.36	0.21–0.60	< 0.01	0.49	0.27–0.88	0.01
**YACD**						
0.00 ~ 1.99 (ref)	1			1		
2.00 ~ 4.99	0.65	0.41–1.04	0.07	0.67	0.41–1.08	0.09
5.00 ~ 9.99	0.71	0.39–1.29	0.26	0.73	0.39–1.38	0.34
≥ 10.00	0.58	0.35–0.96	0.03	0.65	0.37–1.10	0.11

The interaction term (race and smoking status) had statistical significance on subgroups: FS3 (*p* = 0.03) and CS (*p* = 0.01). *NLR* Neutrophil-to-lymphocyte ratio. *CD* Chronic diseases. *BMI* Body mass index. *NHW* Non-Hispanic White. *NHB* Non-Hispanic Black. *NS* Nonsmoker. *FS1* Quit smoking 5 or more years before a cancer diagnosis. *FS2* Quit smoking less than 5 years before a cancer diagnosis. *FS3* Quit smoking after a cancer diagnosis. *CS* Continued smoking after a cancer diagnosis. *RCs* Respiratory cancers. *NRCs* Non-respiratory cancers. *YACD* Years after a cancer diagnosis. *OR* Odds ratio. *CI* Confidence interval.

We selected gender, age, CD free status, BMI, education level, cancer type, and YACD as control covariables for model construction to further explore the racial disparity of NLR risk between NHW and NHB in terms of different smoking statuses (Table 4). After adjusting for all the covariables: NHB individuals who quit smoking less than 5 years before a cancer diagnosis, individuals who quit smoking after a cancer diagnosis, and individuals who continued smoking after a cancer diagnosis were associated with higher odds of high-risk NLR compared to nonsmoker (OR 3.39, 4.83, and 2.84, CIs 1.00–10.45, 1.40–16.66, and 1.11–7.24, *p* = 0.03, 0.01, and 0.03, respectively). NHB survivors who were diagnosed with non-respiratory cancers were associated with lower odds of high-risk NLR, compared to NHB respiratory cancer survivors (OR 0.23, CI 0.07–0.74, *p* = 0.01). For NHW cancer survivors, females had lower odds of high-risk NLR, compared to males (OR 0.62, CI 0.41–0.96, *p* = 0.03). Cancer survivors who identified as NHW age 75 years and above had higher odds of high-risk NLR compared with those younger than 45 years of age (OR 2.83, CI 1.30–6.17, *p* < 0.01). NHW survivors who suffered from chronic diseases had higher odds of high-risk NLR compared to CD free individuals (OR 1.45, CI 1.00–2.10, *p* = 0.05).

**Table 4:** Factors associated with a high-risk NLR across different racial groups.

Characteristic	NHB			NHW		
	Adjusted OR	95% CI	*p* value	Adjusted OR	95% CI	*p* value
**Smoking status**						
NS (ref)	1			1		
FS1	1.55	0.67–3.60	0.28	1.13	0.71–1.81	0.59
FS2	3.39	1.00–10.45	0.03	1.46	0.72–2.96	0.28
FS3	4.83	1.40–16.66	0.01	0.99	0.54–1.82	0.98
CS	2.84	1.11–7.24	0.03	0.83	0.41–1.69	0.62
**Gender**						
Male (ref)	1			1		
Female	0.59	0.26–1.33	0.20	0.62	0.41–0.96	0.03
**Age**						
< 45 (ref)	1			1		
45–54	1.01	0.14–7.20	0.98	1.57	0.54–4.51	0.39
55–64	1.41	0.25–7.76	0.68	1.03	0.41–2.57	0.94
65–74	1.76	0.30–10.24	0.51	1.96	0.87–4.42	0.09
75–85	3.94	0.63–24.65	0.13	2.83	1.30–6.17	< 0.01
**CD Free**						
Yes (ref)	1			1		
No	0.64	0.35–1.20	0.16	1.45	1.00–2.10	0.05
**BMI**						
Under/normal (ref)	1			1		
Overweight/obese	1.69	0.86–3.34	0.12	0.68	0.45–1.03	0.07
**Education Level**						
< High school (ref)	1			1		
≥ High school	0.98	0.52–1.53	0.67	1.06	0.71–1.59	0.74
Cancer types						
RCs (ref)	1			1		
NRCs	0.23	0.07–0.74	0.01	0.52	0.26–1.01	0.06
**YACD**						
0.00 ~ 1.99 (ref)	1			1		
2.00 ~ 4.99	1.22	0.52–2.87	0.63	0.62	0.36–1.06	0.08
5.00 ~ 9.99	0.51	0.16–1.61	0.24	0.78	0.38–1.61	0.51
≥ 10.00	0.54	0.17–1.73	0.29	0.65	0.36–1.17	0.15

*NLR* Neutrophil-to-lymphocyte ratio. *CD* Chronic diseases. *BMI* Body mass index. *NHW* Non-Hispanic White. *NHB* Non-hispanic black. *NS* Nonsmoker. *FS1* Quit smoking 5 or more years before a cancer diagnosis. *FS2* Quit smoking less than 5 years before a cancer diagnosis. *FS3* Quit smoking after a cancer diagnosis. *CS* Continued smoking after a cancer diagnosis. *RCs* Respiratory cancers. *NRCs* Non-respiratory cancers. *YACD* Years after a cancer diagnosis. *OR* Odds ratio. *CI* Confidence interval.

## Discussion

High-risk NLR level is positively correlated with continued cigarette smoking after a cancer diagnosis. This has been consistently reported from clinical epidemiological studies that focused on advocating for smoking cessation for TUR cancers. However, most of these studies exclusively focus on NLR risk level with one cancer type, such as lung^5^ and bladder^6^, or they fail to identify the exact timing of smoking cessation in their statistical models^12^. To explore the hidden association between different smoking cessation times and the risk of increased NLR levels in TUR cancer survivors, we constructed multivariable regression models by utilizing the accumulated NHANES datasets. By setting a fixed cut-off value for NLR level at three, we found the percentage of high-risk NLR in our sample population is 28.2%. This is higher than the percentage with high-risk NLR levels (18.5%) in the general adult population (age ≥ 20 years of age) in the United States when setting the NLR cut-off value as three^40^. Although it is not clear what the hazard ratio between low- and high-risk NLR levels for tobacco use-related cancer survivors in our study is, the observed 1.43 hazard ratio for all-cause mortality when an adult has an NLR level ≥ 3^40^ suggests that further survival analysis is strongly recommended when corresponding mortality data becomes available.

In our study, cancer survivors in the high-risk NLR group (67.7 ± 0.8 years old) were older than the low-risk NLR group (60.2 ± 0.7 years old). This finding is similar to a previous study that examined the association between NLR level and smoking status among 351 adult male prostate cancer survivors with the NHANES 2005–2016 dataset. The mean age of prostate cancer survivors in the high-risk NLR group (≥ 3) is older (73.5 vs. 69.6 years old)^12^. Initially, the individuals who quit smoking less than 5 years before a cancer diagnosis had the highest percent of high-risk NLR level (41.0%), followed by individuals who quit smoking 5 or more years after a cancer diagnosis (35.1%), individuals who quit smoking after a cancer diagnosis (28.7%), and finally current smoker (19.5%). The association between different smoking cessation times and high-risk NLR level disappeared after adjusting for the covariables in the regression models. This could be because race plays a significant role in high NLR levels in terms of different smoking cessation times for selected TUR cancer survivors.

Further stratified multivariable regression analyses observed that for NHB smokers, the highest odds of high-risk NLR were observed in the group who quit smoking less than 5 years before a cancer diagnosis, rather than in the current smoker group. Meanwhile, for NHB smokers who quit smoking 5 or more years before a cancer diagnosis, the increased odds of high-risk NLR compared to NHB nonsmoker was not statistically significant (OR 1.55, CI 0.64–3.60, *p* = 0.28). Although more studies are required to understand why the highest odds of high-risk NLR does not appear among NHB current smoker, setting exactly 5 years as the smoking cessation time allows us to at least identity the smoking cessation time associated NLR risk disparity between NHB and NHW TUR cancer survivors. One source of this disparity could be the increased proportion of female smoker. Considering the overall population, female cancer survivors had lower odds of high-risk NLR in comparison to males, and gender only impacted the risk of high NLR in NHW survivors. Among the NHB survivor population, there were more male current smoker than in the NHW survivor population (22.1% vs. 12.0%, Table 5). The cumulative effects from the covariables of gender^41^ and race^42^ could potentially be the reason why a higher risk of increased NLR level was associated with current smoker in only the NHB population.

**Table 5:** NHB survivors had lower NLR levels than NHW survivors.

Characteristics	NHB	NHW	*p *value
Mean NLR (± SEM)	2.16 (± 0.10)	2.68 (± 0.04)	< 0.01^†^
Mean Age (± SEM), year	61.2 (± 0.9)	62.4 (± 0.6)	0.27^†^
Mean YACD (± SEM), year	8.18 (± 0.4)	10.9 (± 0.4)	< 0.01^†^
**Smoking status by gender**			
Male, n (%)			< 0.01^b^
NS	95 (37.6)	191(33.0)	
FS1	76 (26.6)	235(41.2)	
FS2	16 (6.3)	42 (6.8)	
FS3	19 (7.5)	52 (6.9 )	
CS	54 (22.1)	48 (12.0)	
Female, n (%)			0.23^b^
NS	44 (46.0)	127 (37.0)	
FS1	9 (8.4)	52 (13.8)	
FS2	8 (8.8)	17 (4.8)	
FS3	8 (12.0)	36 (11.8)	
CS	19 (24.9)	115 (32.6)	
Gender, n (%)			< 0.01^a^
Total male	260 (64.5)	568 (53.5)	
Total female	88 (35.5)	347 (46.5)	
Age, n (%)			< 0.01^a^
< 45	25 (12.9)	119 (17.7)	
45–54	30 (15.0)	69 (12.1)	
55–64	94 (25.0)	106 (16.6)	
65–74	110 (25.9)	208 (23.8)	
75–85	89 (21.2)	413 (29.9)	
Cancer types, n (%)			0.91^a^
RCs	20 (6.8)	58 (6.6)	
NRCs	328 (93.2)	857 (93.4)	

^†^One-way ANOVA test found mean age is distributed differently between low-risk and high-risk NLR groups. ^a^Rao-Scott Chi-square test. ^b^Fisher’s exact test. *SEM* Standard error of the mean. *NLR* Neutrophil-to-lymphocyte ratio. *CD* Chronic diseases. *BMI* Body mass index. *NHW* Non-Hispanic White. *NHB* Non-Hispanic Black. *NS* Nonsmoker. *FS1* Quit smoking 5 or more years before a cancer diagnosis. *FS2* Quit smoking less than 5 years before a cancer diagnosis. *FS3* Quit smoking after a cancer diagnosis. *CS* Continued smoking after a cancer diagnosis. *RCs* Respiratory cancers. *NRCs* Non-respiratory cancers.

We found the current smoking rate among all TUR cancer survivors was 21.7%, which was higher than the estimated percentage of current adult cigarette smokers (14.0%) in the general US population^43^. Using cancer diagnosis time as a reference point to stratify cancer survivors with different smoking statuses, a study found that of the 50.6% of cancer survivors who had ever smoked cigarettes before their cancer diagnosis, 36.1% of them quit smoking after their diagnosis with cancer^44^. We optimized this stratification criterion by considering the exact number of years that individuals quit smoking before a diagnosis with a tobacco use-related cancer. The redefined smoking statuses allowed us to find more cancer survivors (64.5%) who smoked cigarettes before a cancer diagnosis. More importantly, only 29.7% of them quit smoking after the diagnosis of a TUR cancer.

Gender, age, race, education level, and cancer type are all potential factors for individuals to quit smoking after a cancer diagnosis^44–46^. A study that used a combined NHANES dataset by examining 566 cancer survivors age ≥ 20 found men were 1.83 times more likely to quit smoking than women after a diagnosis of cancer^44^. We found the highest percentage of women in the current smoker group (66.4%) compared to the other smoking status groups. In the general U.S. population, a higher smoking rate is observed for people younger than 44 years of age^47^. We found the mean age of the current smoker group to be 49.6 years old, and the current smoker group contains the highest percentage of cancer survivors who are younger than 45 years old (39.0%), compared with individuals who quit smoking more than 5 years before a cancer diagnosis (4.6%), individuals who quit smoking 5 years or less before a cancer diagnosis (13.0%), and individuals who quit smoking after a cancer diagnosis (9.7%). These features matched the downward trend of cigarette smoking at the national level^48^. Considering the existence of a racial disparity in smoking cessation between NHW and NHB survivors^49^, we observed a slightly increased percentage of NHB survivors (12.0%) in the current smoking group, compared with NHB survivors who quit smoking after a cancer diagnosis (11.2%). Studies show that cancer patients who have a college degree are nearly twice as likely to quit smoking in comparison to individuals with a high school degree or less^45^. We found a similar trend involving a higher percentage of people who quit smoking after a cancer diagnosis (22.0%) or continued smoking (24.2%) having less than a high school degree compared with individuals who quit smoking 5 or more years before a diagnosis with cancer (19.5%) and individuals who quit smoking less than 5 years before a cancer diagnosis (11.2%).

Compared to non-respiratory cancer survivors, respiratory cancer survivors could potentially feel more guilt and shame as a result of previous smoking^50^. Such negative emotions would significantly lower their smoking cessation motivation after a cancer diagnosis^51^. We found that, among lifetime smokers, respiratory cancer survivors have a lower smoking rate after cancer diagnosis compared to non-respiratory cancer survivors (38.8% vs. 48.8%). The inconsistent findings in this study could potentially be addressed if the corresponding mental health-related data for tobacco use-related cancer survivors becomes available. In addition, more individuals with a higher BMI (≥ 25) were observed in the group that quit smoking after being diagnosed with cancer in comparison to current smoker. This could be because smoking cessation potentially increases food intake by an individual to compensate for the effect of nicotine addiction, which results in individuals who quit smoking having a higher BMI^52,53^. Cumulative smoking cessation time could also potentially positively affect the BMI of former smokers^54^. Therefore, it is reasonable to observe the highest proportion of individuals with BMI ≥ 25 (78.1%) in the cancer survivor group that quit smoking more than 5 years before their cancer diagnosis.

Unfortunately, this study has several limitations. First, NHANES relies on self-reported data and, therefore, bias might be present. For example, the height and weight measurements that are required for computing BMI, and the time of quitting smoking for cancer survivors were all self-reported. Secondly, to get a large enough sample size for this analysis, ten consecutive 2-year survey cycle datasets were combined to reach the necessary statistical power. Such a combination could potentially lead to overcounting the number of participants who were diagnosed with high 5-year survival rate cancers^27^. Third, according to the cancer type, the NLR level could vary both before and after chemotherapy^55^. NHANES does not provide enough information for us to exclude these individuals. Besides, the survival rates for some cancers are extremely low, for example, pancreatic cancer. Additional bias would be introduced if we removed the cancer survivors who had been diagnosed with cancer no more than 2 years ago. Additionally, reducing the high-risk NLR by smoking cessation might not be applicable to hospitalized cancer patients. Finally, findings from this study are based on cross-sectional survey data, therefore, a causal relationship between smoking cessation and NLR cannot be established.

Regardless of the limitations, this study has three major strengths. It first combined fourteen well-documented TUR cancer types from a national survey dataset and tried to identify the association between smoking status and the risk of high NLR levels. Then, it defined the exact number of years since smoking cessation relative to the time of cancer diagnosis to study the association between different smoking cessation times and the risk of high NLR levels. It also further captured the racial disparity that regulates the high-risk NLR among NHB cancer survivors exclusively. This study not only confirms that NHB cancer survivors who continued smoking cigarettes are at a higher high-risk NLR level^12^, but also revealed that smoking cessation time and length are associated with a high risk of high NLR levels for TUR cancer survivors.

## Conclusion

Our study found that NHB TUR cancer survivors who quit smoking after a cancer diagnosis were likely to have the highest risk of high NLR levels, which was followed by those who quit smoking less than 5 years before a cancer diagnosis and current smoker when compared with nonsmoker. These findings suggest that risk of a high NLR level is strongly associated with smoking cessation time in NHB TUR cancer survivors. Therefore, NHB TUR cancer survivors should quit smoking as soon as possible because the benefits of quitting smoking were most noticeably observed when survivors had been smoke-free for more than 5 years.

In future studies, identifying potential barriers that prevent older NHW(age 75 years above) from quitting smoking after diagnosis of TUR cancers are strongly recommended. Meanwhile, the smoking cessation intervention programs should pay extra attention to current NHB smokers who undergo the cancer screening programs.

## Data Availability

Publicly available datasets were analyzed in this study. This data can be found at: https://www.cdc.gov/nchs/nhanes/index.htm.
